# Analysis of Cost and Treatment Effects in the Care Given for Graves' Disease: A Swedish Cost–Utility Analysis

**DOI:** 10.1002/edm2.70034

**Published:** 2025-02-21

**Authors:** Lars Lindholm, Gabriel Sjölin, Annika Jonsson, Mirna Abraham‐Nordling, Göran Wallin, TT‐12 Study Group, Helena Filipsson Nyström, Christoffer Andersén, Jan Calissendorff, Lovisa Ekestubbe, Helena Filipsson Nyström, Klara Gewert, Bengt Hallengren, Mats Holmberg, Selwan Khamisi, Mikael Lantz, Daniel Mauritzson, Tereza Planck, Gabriel Sjölin, Ruzan Udumyan, Jeanette Wahlberg, Göran Wallin, Ove Törring

**Affiliations:** ^1^ Department of Epidemiology and Global Health Umeå University Umeå Sweden; ^2^ Faculty of Medicine and Health Örebro University Örebro Sweden; ^3^ Department of Surgery Örebro University hospital Örebro Sweden; ^4^ Department of Health Planning Region Jämtland Härjedalen Östersund Sweden; ^5^ Department of Molecular Medicine and Surgery Karolinska Institutet Stockholm Sweden; ^6^ National Task Force for Hyperthyroidism Swedish Knowledge Organisation, Region Västerbotten Umeå Sweden; ^7^ Institute of Medicine, Sahlgrenska Academy, University of Gothenburg Göteborg Sweden; ^8^ Department of Endocrinology Sahlgrenska University Hospital Göteborg Sweden; ^9^ Wallenberg Centre for Molecular and Translational Medicine University of Gothenburg Göteborg Sweden

**Keywords:** cost‐effectiveness analysis, Graves' disease, health economics, ICER, QALY

## Abstract

**Background:**

Guidelines in healthcare should be evidence‐based, satisfy patient needs and improve patient outcome.

**Methods:**

We performed a cost–utility analysis in Graves' disease (GD) and estimated incremental costs after the introduction of a national guideline adding the Graves' Recurrent Events After Therapy (GREAT) score with genetic determinants (GREAT+) to predict recurrence, a thyroid nurse, preoperative calcium/vitamin D treatment and thyroid‐stimulating immunoglobulins.

**Findings:**

Antithyroid drugs (ATDs) were less costly, achieved 0.88 quality‐adjusted life years (QALYs) over 8 years and dominated over radioactive iodine (RAI) treatment. The relevant incremental cost‐effectiveness ratio was ATD versus thyroid surgery (Tx). Tx was more costly than ATD but was also more effective. The incremental cost‐effectiveness ratio was equal to 40,488 Euro per QALY gained. In recurrent GD, the QALY weight for surgery after ATD was 0.76 compared with 0.79 when surgery was the initial treatment. If individuals requiring surgery could be identified at start of first treatment, QALYs would be higher (6.32) and the cost lower (13,945 Euro). The net cost increase after the new guideline was 17.6%, which was partially an effect from more time being spent with the thyroid nurse. If the GREAT+ score was also applied, the total increased net cost was 14.8% if 24% of the tested patients changed treatment to Tx.

**Interpretation:**

Tx was more cost‐effective than RAI when ablative treatment is advocated. Prediction score for recurrence directing patients to earlier Tx is cost‐effective and enables the introduction of a specialist thyroid nurse. Health economic evaluations should accompany future guidelines.

## Graphical abstract

Surgery was more cost‐effective than RAI when ablative treatment is advocated. Prediction score for recurrence directing patients to earlier Tx is cost‐effective and enables the introduction of a specialist thyroid nurse. Health economic evaluations should accompany future guidelines.

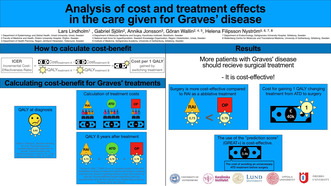

## 1 Introduction

Progress in medical science can lead to increased healthcare costs. If new evidence‐based practices are evaluated for cost‐effectiveness, healthcare resources can be used more efficiently. International guidelines are fundamental for evidence‐based recommendations, but costs and healthcare systems differ by country. Sweden has a tax‐financed healthcare system with the goal of providing accessible, equal and harmonised healthcare for all.

The Swedish ‘Knowledge Organisation’ aims to harmonise care and requires patient involvement in its task forces to better adapt care to correspond with patient needs [1]. Hyperthyroidism has been identified as an area where practice needs to be more consistent and the task force for the national guideline decided to include health economic aspects for Graves' disease (GD), as patients with GD need to choose between antithyroid drug (ATD), thyroidectomy (Tx) or radioactive iodine (RAI) treatments. This choice will be influenced by the pros and cons of treatment [2, 3], risk of recurrent disease, and fears and personal preferences of physicians and patients. Should the cost of the treatment also be included as a variable?

After first‐time ATD treatment, the disease recurrence rate is 54.7% [4], even if many patients with the most severe forms of the disease are directed to Tx or RAI early in treatment course [4]. This is in accordance with European practice [5, 6]. Would a score that gives patients a more thorough risk estimate [7] be cost‐effective if it changed patient preference of treatment?

A well‐established risk assessment tool for recurrence in antithyroid drug therapy is the Graves' Recurrent Events After Therapy (GREAT) score [7]. It classifies patients into three risk groups for recurrence, utilising common clinical markers (age, s‐fT4, s‐TBII and goitre size). With the GREAT+ score [7], also including addition of genetic markers (HLA polymorphisms and PTPN22), the precision of predicting treatment response can be further enhanced, particularly in the intermediate risk group, with recurrence risks ranging from 4% in Class I+ to 84% in Class IV+.

In addition, patients have highlighted the need for more information, better accessibility, quality of life (QoL) measurement and a rehabilitation care process if they do not recover as fast as expected. Mental symptoms persist in as many as 38% of premenopausal women after GD treatment [8, 9]. In the national guideline, we therefore recommended a specialist thyroid nurse which may increase accessibility, manage QoL measurements and contribute to a team‐based way of working. Contact nurses have been deemed cost‐effective in emergency healthcare [10] and in the treatment of other diseases [11, 12]. How would that affect cost‐effectiveness in our setting?

In September 2020, the literature was searched for cost–utility and cost‐effectiveness analyses in GD comparing at least two of three outcomes of ATD, Tx or RAI treatments, information on *quality*‐adjusted life years (*QALYs*), and possibly the incremental cost‐effectiveness ratio (ICER). Three manuscripts were identified as adequate [13, 14, 15], of which the full text of each was read and evaluated according to the Swedish national guidelines for quality and transferability for health economic studies [16]. None of these studies was considered suitable to be used in a Swedish context.

Instead of modelling costs and outcomes, we used data from two Swedish studies [17, 18] to perform a cost–utility analysis with the aims of (1) estimating which treatment choice was the most cost‐effective before the implementation of the national guidelines and (2) estimating incremental costs and incremental QALYs after the introduction of the guidelines when adding the GREAT+ score to predict recurrence [7], a thyroid nurse, preoperative calcium/vitamin D treatment and thyroid‐stimulating immunoglobulins (TSIs). We estimate to use TSIs in a minority of TSH receptor antibody (TRAb)‐negative cases in addition to scintigraphy, as TSI analysis has better sensitivity and specificity than TRAb analysis [19].

## 2 Methods

### 2.1 Real‐Life QALY Data at Graves' Disease Diagnosis

TT‐96 is a randomised, controlled trial of the outcome of ATD and RAI on the risk for Graves' orbitopathy in 313 GD patients at diagnosis [17]. We used the baseline QoL data to calculate QALY weights at baseline. TT‐12 study is the largest follow‐up study of GD in the world comprising 1186 patients. It is an observational study where patients were evaluated 8 years after first‐time GD diagnosis [4, 18]. TT‐12 originates from an incidence population from 2003 to 2005 [20]. We used the 36‐Item Short Form survey (SF‐36) [21] QoL questionnaire to calculate QALY weights after treatment for patients undergoing usual care [22, 23]. The difference in QALY levels between treatments was validated using a 95% confidence interval and a *t*‐test to ensure a significant difference.

### 2.2 Calculation of Costs

The costs for usual care before and after implementation of the national guideline were calculated per treatment (Table 1). Before the guideline, this included costs for ATD, Tx, RAI, laboratory measurements, thyroid scintigraphy, visits and direct treatment‐related costs (Appendix S2). The costs after the introduction of the national guideline included fewer liver and white blood tests, calcium/vitamin D treatment in those with low 25‐OH vitamin D levels before surgery, and GREAT+ score used in the subgroup of patients with moderate recurrence risk according to the GREAT score as suggested by Vos and colleagues [7]. We estimated that the patients who were classified with high risk for recurrence in the GREAT+ score would choose the most cost‐effective ablative treatment and those with the lowest risk would choose ATD. The extra cost for the thyroid nurse was estimated to three extra hours per patient (Table 2) and, as each patient underwent 1.54 treatments, we calculated 1.9 extra hours per treatment round (Table 1). In addition, QoL assessment and TSIs in 1% of patients were included (Appendix S3), which reduced the number of thyroid scintigraphies from 5% to 3%. As a considerable number of patients had GD relapse [4], costs were also calculated per patient (Table 2). The exchange rate on 22 March 2022 for 100 Swedish krona (SEK) was 9.48 United States dollars (USD) and 10.42 Euros.

**TABLE 1:** Calculations of cost before and after introduction of the national guideline per treatment episode.

	Cost							
	Average cost of treatment before introduction of new guideline	New average cost of genetic analysis in GREAT+ score (50% of patients)	New costs of visit to thyroid nurse	Change in costs of laboratory schedule and drugs	New average cost of TSI (1% of patients at start)	Change in cost of scintigraphy	Average cost of treatment after introduction of new guideline	Cost increase
In SEK								
ATD^a^	37,216	4250	6691	−312	2	−69	47,779	10,563
RAI	50,406	4250	6691	−5	2	0	61,345	10,939
Tx	138,486	4250	6691	219	2	−69	149,580	11,094
In USD								
ATD^a^	3927	448	706	−33	0·2	−7	5041	1115
RAI	5319	448	706	−1	0·2	0	6473	1154
Tx	14,612	448	706	23	0·2	−7	15,783	1171
In Euro								
ATD^a^	3571	408	642	−30	0·2	−7	4585	1014
RAI	4837	408	642	0	0·2	0	5886	1050
Tx	13,288	408	642	21	0·2	−7	14,353	1064

Abbreviations: ATD, antithyroid drug; GREAT, Graves' Recurrent Events After Therapy; RAI, radioactive iodine; SEK, Swedish krona; TSI, thyroid‐stimulating immunoglobulin; Tx, thyroidectomy; USD, United States dollar.

^a^ Average block and replace monotherapy.

**TABLE 2:** Calculations of cost before and after introduction of the national guideline per patient including > 1 treatment.

	Cost							
	Average cost of treatment before introduction of new guideline	Genetic analysis in GREAT+ score (50% of patients)	Visit to thyroid nurse	Laboratory schedule and drugs	New average cost of TSI (1% of patients at start)	Change in cost of scintigraphy	Average cost of treatment after introduction of new guideline	Cost increase
In SEK								
ATD^a^	40,723	4250	7498	−356	2	−69	52,048	11,325
RAI	68,192	4250	10,803	−190	2	0	83,057	14,865
Tx	164,660	4250	13,305	−132	2	−69	182,016	17,356
In USD								
ATD^a^	4297	448	791	−38	0·2	−7	5492	1195
RAI	7195	448	1140	−20	0·2	0	8764	1568
Tx	17,374	448	1404	−14	0·2	−7	19,205	1831
In Euro								
ATD^a^	3907	408	719	−34	0·2	−7	4994	1087
RAI	6543	408	1037	−18	0·2	0	7970	1426
Tx	15,800	408	1277	−13	0·2	−7	17,465	1665

Abbreviations: ATD, antithyroid drug; GREAT, Graves' Recurrent Events After Therapy; RAI, radioactive iodine; SEK, Swedish krona; TSI, thyroid‐stimulating immunoglobulin; Tx, thyroidectomy; USD, United States dollar.

^a^ Average block and replace monotherapy.

### 2.3 ICER Calculation

All interventions were ranked according to their cost. We then took the increment in costs and QALYs between the second and the first intervention and divided the increment in cost with the increment in QALYs, which is equal to the ICER. This calculation was repeated for the second and third interventions. If a certain intervention showed higher cost and lower effectiveness than the previous in the sequence, this intervention was excluded due to dominance. The ICER for the interventions neighbouring the excluded one was recalculated after the exclusion.

### 2.4 Role of Funding Sources

None of the funding sources was involved in any part of this research project.

## 3 Results

### 3.1 Cost‐Effectiveness of Treatments for Graves' Disease Before the New National Guideline

At the time point of first treatment, the alternatives are mutually exclusive. ATD was less costly and achieved 0.88 QALYs over 8 years. RAI was more costly than ATD but had a lower effectiveness in terms of QALYs (0.64) (Table 3). Thus, ATD dominated over RAI and the relevant ICER was ATD versus Tx. Tx was more costly than ATD but was also more effective, and the ICER was equal to 40,488 Euro (44,522 USD, 421,960 SEK) per QALY gained (Table 3). The current threshold value in Sweden is 48,000 Euro (53,000 USD, 500,000 SEK) per QALY gained [24], and Tx is consequently a cost‐effective treatment.

**TABLE 3:** Incremental QALYs and costs gained.

	QALY data			
	QALY weight before treatment	QALY weight after 8 years (95% CI)	QALYs gained over 8 years	Incremental QALYs
ATD	0·65	0·76 (0·75–0·77)^a^	0.88	0.88
RAI	0·65	0·73 (0·72–0·75)^a^	0.64	—
Tx	0·65	0·79 (0·76–0·83)^a^	1.12	0.24

	Cost data		
	Treatment costs over 8 years	Incremental cost	ICER
In SEK			
ATD	37,216	37,216	42,291
RAI	50,406	13,190	—
Tx	138,486	101,270	421,960
In USD			
ATD	3927	3927	4462
RAI	5319	1392	—
Tx	14,612	10,685	44,522
In Euro			
ATD	3571	3571	4058
RAI	4837	1266	—
Tx	13,288	9717	40,488

Abbreviations: ATD, antithyroid drug; ICER, incremental cost‐effectiveness ratio; QALY, quality‐adjusted life year; RAI, radioactive iodine; SEK, Swedish krona; Tx, thyroidectomy; USD, United States dollar.

a *t*‐tests show statistically significant differences (*p* < 0.05) in all comparisons between treatments (ATD vs. RAI, RAI vs. Surgery and ATD vs. surgery).

However, the treatments over time are not mutually exclusive. A significant proportion (54.7%) [4] of those initially treated with ATD need further treatments, either Tx or RAI, due to recurrent disease. Such a case is described in Table 4. The initial treatment was ATD with remission after 2 years. In year 3, symptoms reappeared, and surgery was necessary in year 4. The QALY weight for Tx after ATD was 0.76, thus lower than the weight achieved when surgery is the initial treatment (0.79). The total number of QALYs gained over 8 years was 5.97 and the total cost is equal to 18,122 Euro (19,927 USD, 188,859 SEK).

**TABLE 4:** Change in cost and QALYs gained when GREAT+ score is implemented.

	Year									
	0	1	2	3	4	5	6	7	8	Total (1–8)
*ATD year 1 + Tx year 4*										
QALYs gained	0·65	0·76	0·76	0·65	0·76	0·76	0·76	0·76	0·76	5·97
ATD cost in SEK	—	43,529	—	—	—	—	—	—	—	—
ATD cost in USD	—	4593	—	—	—	—	—	—	—	—
ATD cost in Euro	—	4177	—	—	—	—	—	—	—	—
Tx cost in SEK	—	—	—	—	145,330	—	—	—	—	—
Tx cost in USD	—	—	—	—	15,334	—	—	—	—	—
Tx cost in Euro	—	—	—	—	13,945	—	—	—	—	—
*Tx year 1*										
QALYs gained	0·65	0·79	0·79	0·79	0·79	0·79	0·79	0·79	0·79	6·32
Tx cost in SEK	—	145,330	—	—	—	—	—	—	—	—
Cost GREAT+ score^a^ in SEK	—	4250	—	—	—	—	—	—	—	—
Cost GREAT+ score^b^ in SEK	—	35,417	—	—	—	—	—	—	—	—
Tx cost in USD	—	15,334	—	—	—	—	—	—	—	—
Cost GREAT+ score^a^ in USD	—	897	—	—	—	—	—	—	—	—
Cost GREAT+ score^b^ in USD	—	3737	—	—	—	—	—	—	—	—
Tx cost in Euro	—	13,945	—	—	—	—	—	—	—	—
Cost GREAT+ score^a^ in Euro	—	816	—	—	—	—	—	—	—	—
Cost GREAT+ score^b^ in Euro	—	3398	—	—	—	—	—	—	—	—

Abbreviations: ATD, antithyroid drug; ICER, incremental cost‐effectiveness ratio; QALY, quality‐adjusted life year; SEK, Swedish krona; Tx, thyroidectomy; USD, United States dollar.

^a^ Cost for each GREAT+ score.

^b^ Cost of GREAT+ score per changed treatment.

### 3.2 Estimating Costs/QALY Following the National Guideline

If those individuals who require Tx after ATD could be identified earlier in the process, before the initial treatment is chosen, the number of QALYs would be higher (6.32) and the cost lower (13,945 Euro, 15,334 USD, 145,330 SEK). In our analysis (Table 4), the cost for genetic sampling in the GREAT+ score was not included. If estimating its cost at 816 Euro (897 USD, 8500 SEK) (Table 4) and that not all those tested change their treatment, we assume that for 100 tests, 24 patients [4] will change the choice of initial treatment from ATD to Tx: the estimated cost per changed treatment would be 3398 Euro (3737 USD, 35,417 SEK). Using the GREAT+ score could thus prevent repeated treatments, increase the gained QALYs and lower overall treatment costs.

Estimating the cost from the national guideline by adding the cost for a thyroid nurse, and the cost of the GREAT+ score as major costs (Appendix S3), the net cost increase was 1014–1064 Euro (1115–1171 USD, 10,563–11,094 SEK) depending on treatment choice (Table 1).

The net cost after the new guideline was 17.6% higher, which was partially an effect from more time spent with the patient by the thyroid nurse. If a change in strategy for the individual choice according to the GREAT+ score is also taken into account, the total net cost before and after the national guideline was 14.8% higher if 24% of the tested patients changed treatment to Tx [4].

## 4 Discussion

ATD was the cheapest therapy, but the effectiveness of Tx was higher: the ICER for Tx compared with ATD was below the Swedish threshold. Thus, there is no reason to ration surgery from the cost‐effectiveness point of view. However, the cost‐effectiveness of Tx on recurrent GD after a treatment course with ATD is lower than Tx at an earlier stage. If those individuals who require ablative treatment after ATD could be identified earlier in the process, that is, before the initial treatment was chosen, the number of QALYs would be higher and the cost lower if surgery was chosen earlier. Hence, the GREAT+ score was cost‐effective even if only about one‐quarter of the patients changed their treatment [4].

The first lesson to learn from this study is that Tx may be chosen before RAI in situations when ablative treatment is preferred, and there are no individual or medical reasons to choose a non‐surgical strategy, as it is the most effective and even cost‐effective treatment to protect from recurrent disease. This has also been shown in a recent study by Ma and colleagues [25]. In our study, we have not included the cost for complications from surgery, such as hoarseness due to a laryngeal nerve palsy, hypocalcaemia due to permanent damage of the parathyroid glands [2], the cost for an increased frequency of Graves' orbitopathy after RAI [17, 26] or side effects of ATD [2]. However, their effect on quality of life is included in QALY calculation. If Tx is undertaken during day‐time care instead of an overnight stay in hospital [27], the cost will most likely become lower over time. Hence, cost‐effectiveness is a criterion to take into consideration for the next national guideline. To expand the use of Tx would certainly increase outcomes in terms of health‐related QoL, especially in countries where there is a good availability of high‐volume surgeons with a relatively low frequency of complications. The introduction of calcium/vitamin D treatment preoperatively to reduce the temporary hypocalcaemia postoperatively [28] will further underline the safety of Tx in cases with a high GD recurrence rate. Also, the effect probably lasts much longer than the 8 years we considered in our study [4]. If we assume a life‐long effect, that is, a further 30 years, the cost per QALY drops significantly to around 2498 EURO (2747 USD, 26,000 SEK). This means that our calculations certainly overestimate the cost per QALY gained.

A cost‐effectiveness ratio is difficult to interpret—it is just a ‘price’ necessary to pay for achieving a QALY gain. We thus need to decide whether we, as a society, are willing to pay this price. The societal willingness to pay is known as the ‘threshold value’ and differs between countries [29]. The threshold value in Sweden is 48,000 Euro (53,000 USD, 500,000 SEK) per QALY gained, as mentioned earlier. This can be interpreted as a rule of thumb and has been established through decisions by responsible Swedish authorities such as the Dental and Pharmaceutical Benefits Agency and the Board for Health and Welfare [24]. This threshold is approximately at the same level as other countries in Northwest Europe [24, 29] and means that a treatment with a cost under the threshold is to be seen as cost‐effective. National factors, including differences in treatment access and cost disparities, further complicate cost‐effectiveness analysis. This is exemplified in a recent Ethiopian study [30], which contradicts our results by showing non‐inferiority of RAI compared with ATD. In their context, ATD carries a higher cost than RAI, accentuating the intricacies of such analyses.

The second lesson to learn is that the GREAT+ score [7], which targets precision medicine with an individual risk calculation for recurrent disease, was cost‐effective. A prerequisite for this reasoning is that patients will change their choice of treatment if they are provided with information on a low versus high risk for recurrent disease. This was explored by van Kinschot and colleagues [31] who concluded that the risk of recurrent disease is the most important factor explaining 37% of a patient's choice. As recurrent disease after ATD treatment occurs in approximately 50% of cases [4, 7, 32, 33], individualising the risk to 16% or 68% [7] will probably matter for patients.

As of today, patients with high thyroid hormone levels, high TRAbs and/or large goitres are more prone to ablative treatment, but implementing the GREAT+ score consecutively has been validated retrospectively, ranging from a recurrence rate of 33.8% in class I to 73.8% in class III in a Swiss population [32]. According to Vos and colleagues [7], the group benefiting from the GREAT+ score is those in class II with 44% recurrence risk where genotype analyses change recurrence risk in 38% of cases. In most cases, the GREAT+ score provided a lower risk estimate and therefore advocates for ATD therapy, whereas in 5% of cases genotyping provided a very high recurrence rate. In the calculations in our study, we used a change of treatment towards Tx for 24% of the patients for whom genotype analyses indicated a high or moderate recurrence rate. Use of predictive scores has several advantages. First, patients receive better information about treatments and recurrence risks, and thereby a better understanding. This will likely lead to better treatment compliance and thereby lower recurrence rates. A lower rate of disease recurrence will lower treatment costs.

The third lesson and novelty in our study is that we used retrospective real‐life data instead of modelling effects and costs [13, 14, 15]. The latter is the most common approach in cost‐effectiveness analyses. Previous modelling reports on GD treatment advocate Tx as most cost‐effective [14, 15], although one group [13] states cost‐effectiveness for RAI [13]. Both approaches have pros and cons. Retrospective real‐life cost and effectiveness data generated in the same setting give accurate information during the follow‐up window. However, it is not unusual that effects last beyond this window, as in the case of Tx. If these effects beyond the window are not included, the analysis will be biased because all costs are typically included, since they appear within the trial period. Thus, a strength of the modelling approach is the ability to estimate future effects and even costs. However, the uncertainty in prognosis can be significant when data generated in different settings are combined.

The fourth lesson to learn is that a health economic evaluation before the introduction of a national guideline can be used as a motivation for improvements for patients. Implementing a specialised nurse has proven cost‐effective in other diseases [10, 11, 12, 13, 34] and is considered the improvement that will benefit patients the most: however, at the same time, it is the action that costs the most. We can calculate the cost for the new national guideline, but we cannot estimate its full effect in QALYs. Nevertheless, we can argue that the cost for the guideline will be lower if, at the same time, it contributes to a more cost‐effective choice of treatment, leads to lower complication frequency and results in a better path forward for patients through the healthcare system with rehabilitation actions for those that do not recover as expected. This has recently been suggested in a review for patients with hypothyroidism [35]. In addition, even if the cost is small compared with a thyroid nurse, TSI analyses are superior to TRAb in hyperthyroidism [19] and may prevent some of the later costs for thyroid scintigraphies. However, the time is not right to replace TRAb with TSIs in hyperthyroidism in Sweden, as no laboratory has TSI measurement available. As far as we know, no one has estimated intervention costs before and after a national guideline within thyroid diseases, even though hyperthyroidism is frequent in the population [20].

We would like to hold forward the following strengths: our study has been performed using real‐life data instead of modelling and that estimates are based on the development of a national guideline. It is based on the costs for regular visits, laboratory and drugs, but neither scenarios of complications nor estimates that the national guideline changes patient flows have been added in the calculation of costs. Even though the GREAT+ score is established in a Dutch population, which is not very different from a Swedish population, it is hard to estimate the effect on patient flows, as the patient's perspective includes more than considering the recurrence rate [31]. Yet, we can estimate that changing patient flow to earlier Tx in those that will have a recurrent disease will save costs, which can be used to develop the concept of a thyroid nurse, which will most likely benefit patient outcome in the short‐ and long‐term perspective, as shown for other diseases [10, 11, 12, 34].

The Swedish way of managing hyperthyroid patients is similar to that in Europe [5, 6]. Consequently, the lessons learned in this study can be adopted in other Western countries as well. Health economics is an aspect to take into consideration in meetings with GD patients. GD is consequently a disease where we can work more cost‐effectively to provide care that may further improve outcome. The use of RAI has decreased in Europe over the last two decades in favour of ATD therapy [6], and some authors advocate a second treatment period in case of recurrent disease [36]. RAI is still frequent in the United States [37]. A health economic evaluation is a product of its system. In a tax‐financed system such as in Sweden, the continuous advances in medicine will always be underfinanced and money needs to be used as effectively as possible.

## Author Contributions

L.L., G.S., A.J., G.W. and H.F.N. made a substantial contribution to the concept or design of the article, and the acquisition, analysis and interpretation of data for the article. M.A.‐N. made a substantial contribution to the acquisition of data for the article. M.A.‐N. and G.W. revised the article critically for important intellectual content. L.L., G.S., A.S. and H.F.N. drafted the article. All authors approved the version to be published and agreed to be accountable for all aspects of the work in ensuring that questions related to the accuracy or integrity of any part of the work are appropriately investigated and resolved.

## Conflicts of Interest

The authors declare no conflicts of interest.

## Supporting information


Data S1.

## Data Availability

Patient data availability is not applicable to this article as no new data were created or analysed in this study. The economic data that support the findings of this study are available within the article and its appendix.
