# Supplementary material for: Analysis of Cost and Treatment Effects in the Care Given for Graves' Disease: A Swedish Cost–Utility Analysis
Source: Endocrinol Diabetes Metab. 2025 Feb 21;8(2):e70034. doi: 10.1002/edm2.70034 (PMC11844766; doi:10.1002/edm2.70034)
Supplement: Supplementary file 1 — Data S1. [file EDM2-8-e70034-s001.pdf]

Appendix

Table S1: Allocation of costs on treatments before introduction of new guideline..............................2

Table S2: Allocation of costs on treatments after introduction of new guideline..................................3

Table S3: Number of laboratory tests before and after introduction of new guideline.........................4

Table S4: Average cost of laboratory tests before and after introduction of new guideline in SEK......5

Table S5: Average cost of laboratory tests before and after introduction of new guideline in USD.....6

Table S6: Average cost of laboratory tests before and after introduction of new guideline in Euro.....7

Table S7: Estimated new changes after introduction of new guideline..8

Item	Cost		
	ATD*	RAI	Tx
In SEK			
Laboratory tests	2418	1983	1613
Doctor visits at medical clinic	26 115	20 892	10 446
Drugs	6258	1586	6322
Scintigraphy†	173	3462	173
Thyroid ultrasound	0	0	2300
RAI treatment	0	6237	0
Tx	0	0	94 149
Visits to surgeon pre- and post-Tx	0	0	14 100
Thyroxine‡	2252	16 246	9383
**TOTAL per treatment**	**37 216**	**50 406**	**138 486**
In USD			
Laboratory tests	255	209	170
Doctor visits at medical clinic	2755	2204	1102
Drugs	660	167	667
Scintigraphy†	18	365	18
Thyroid ultrasound	0	0	243
RAI treatment	0	658	0
Tx	0	0	9934
Visits to surgeon pre- and post-surgery	0	0	1488
Thyroxine‡	238	1715	990
**TOTAL per treatment**	**3927**	**5319**	**14 612**
In Euro			
Laboratory tests	232	190	155
Doctor visits at medical clinic	2506	2005	1002
Drugs	600	152	607
Scintigraphy†	17	332	17
Thyroid ultrasound	0	0	221
RAI treatment	0	598	0
Tx	0	0	9034
Visits to surgeon pre- and post-surgery	0	0	1353
Thyroxine‡	216	1559	900
**TOTAL per treatment**	**3571**	**4837**	**13 288**

*Average block and replace monotherapy. †In 5% of patients at first visit and all RAI (cost 3462 SEK (365 USA, 332 Euro) per scintigraphy. ‡Average life-long cost for patients not becoming euthyroid. ATD, antithyroid drug; RAI, radioactive iodine; SEK, Swedish krona; Tx, thyroidectomy; USD, United States dollar.

Table S1: Allocation of costs on treatments before introduction of new guideline

Item	Cost		
	ATD*	RAI	Tx
In SEK			
Laboratory tests	2109	1980	1758
GREAT+ score†	4250	4250	4250
Doctor visits at medical clinic	26 115	20 892	10 446
Contact nurse visits	6691	6691	6691
Drugs	6258	1586	6306
Calcium/vitamin D treatment‡	0	0	93
Scintigraphy¶	104	3462	104
Thyroid ultrasound	0	0	2300
RAI treatment	0	6237	0
Tx	0	0	94 149
Visits to surgeon pre- and post-Tx	0	0	14 100
Thyroxine§	2252	16 247	9383
**TOTAL per treatment**	**47 779**	**61 345**	**149 580**
In USD			
Laboratory tests	223	209	185
GREAT+ score†	448	448	448
Doctor visits at medical clinic	2755	2204	1102
Contact nurse visits	706	706	706
Drugs	660	167	665
Calcium/vitamin D treatment‡	0	0	10
Scintigraphy¶	11	365	11
Thyroid ultrasound	0	0	243
RAI treatment	0	658	0
Tx	0	0	9934
Visits to surgeon pre- and post-surgery	0	0	1488
Thyroxine§	238	1714	990
**TOTAL per treatment**	**5041**	**6473**	**15 783**
In Euro			
Laboratory tests	202	190	169
GREAT+ score†	408	408	408
Doctor visits at medical clinic	2506	2005	1002
Contact nurse visits	642	642	642
Drugs	600	152	605
Calcium/vitamin D treatment‡	0	0	9
Scintigraphy¶	10	332	10
Thyroid ultrasound	0	0	221
RAI treatment	0	598	0
Tx	0	0	9034
Visits to surgeon pre- and post-surgery	0	0	1353
Thyroxine§	216	1559	900
**TOTAL per treatment**	**4585**	**5886**	**14 353**

*Average block and replace monotherapy. †In 50% of patients (cost 8500 SEK, 897 USD, 816 Euro) per analysis. ‡Assuming 25% of patients with low values. ¶In 3% of patients at first visit and all RAI (cost 3462 SEK, 365 USD, 332 Euro) per scintigraphy. §Average life-long cost for patients not becoming euthyroid. ATD, antithyroid drug; GREAT, Graves' Recurrent Events After Therapy; RAI, radioactive iodine; SEK, Swedish krona; Tx, thyroidectomy; USD, United States dollar.

Table S2: Allocation of costs on treatments after introduction of new guideline

Laboratory test	No. of tests			
	ATD monotherapy	ATD block and replace	RAI	Tx
**Before introduction of new guideline**				
FT4, TSH	14	11	11	7
Haemoglobin, thrombocytes	1	1	1	2
Leucocytes/neutrophils	11	9	4	5
Liver tests	11	9	4	5
Sodium	1	1	1	2
Potassium	1	1	1	2
Creatinine	1	1	1	2
Calcium	1	1	1	4
TRAb	4	4	4	1
FT3	1	1	2	2
Calcium (corrected)	0	0	0	3
PTH	0	0	0	2·14
**After introduction of new guideline**				
FT4, TSH	16	13	11	7
Haemoglobin, thrombocytes	1	1	1	2
Leucocytes neutrophils	3	2	3	3
Liver tests	3	2	3	3
Sodium	1	1	1	2
Potassium	1	1	1	2
Creatinine	1	1	1	2
Calcium	1	1	1	4
HbA1c	1	1	1	1
TRAb	4	4	4	1
TSI (in 1% of patients)	0–1	0–1	0–1	0–1
FT3	1	1	2	2
Calcium (corrected)	0	0	0	3
25-OH-vitamin D3	0	0	0	1–2
PTH	0	0	0	3

FT3, free triiodothyronine; FT4, free thyroxine; HbA1c, glycated haemoglobin; PTH parathyroid hormone; TRAb, anti-TSH receptor antibody; TSH thyroid-stimulating hormone; TSI, thyroid-stimulating immunoglobulin.

Table S3: Number of laboratory tests before and after introduction of new guideline

Laboratory test	Average cost of tests (SEK)			
	ATD monotherapy	ATD block and replace	RAI	Tx
**Before introduction of new guideline**				
FT4, TSH	812	638	638	406
Haemoglobin, platelets	26	26	26	52
Leucocytes/neutrophils	132	108	48	60
Liver tests	583	477	212	265
Sodium	14	14	14	28
Potassium	14	14	14	28
Creatinine	14	14	14	28
Calcium	13	13	13	52
TRAb	920	920	920	230
FT3	42	42	84	84
Calcium (corrected)	0	0	0	132
PTH	0	0	0	248
**TOTAL cost**	**2570**	**2266**	**1983**	**1613**
**After introduction of new guideline**				
FT4, TSH	928	754	638	406
Haemoglobin, platelets	26	26	26	52
Leucocytes neutrophils	36	24	36	36
Liver tests	159	106	159	159
Sodium	14	14	14	28
Potassium	14	14	14	28
Creatinine	14	14	14	28
Calcium	13	13	13	52
HbA1c	60	60	60	60
TRAb	920	920	920	230
TSI (in 1% of patients)	2	2	2	2
FT3	42	42	84	84
Calcium (corrected)	0	0	0	132
25-OH-vitamin D3	0	0	0	213
PTH	0	0	0	248
**TOTAL cost**	**2228**	**1989**	**1980**	**1758**

FT3, free triiodothyronine; FT4, free thyroxine; HbA1c, glycated haemoglobin; PTH parathyroid hormone; SEK, Swedish krona; TRAb, anti-TSH receptor antibody; TSH thyroid-stimulating hormone; TSI, thyroid-stimulating immunoglobulin.

Table S4: Average cost of laboratory tests before and after introduction of new guideline in SEK

Laboratory test	Average cost of tests (USD)			
	ATD monotherapy	ATD block and replace	RAI	Tx
**Before introduction of new guideline**				
FT4, TSH	86	67	67	43
Haemoglobin, platelets	3	3	3	5
Leucocytes/neutrophils	14	11	5	6
Liver tests	62	50	22	28
Sodium	1	1	1	3
Potassium	1	1	1	3
Creatinine	1	1	1	3
Calcium	1	1	1	5
TRAb	97	97	97	24
FT3	4	4	9	9
Calcium (corrected)	0	0	0	14
PTH	0	0	0	26
**TOTAL cost**	**271**	**239**	**209**	**170**
**After introduction of new guideline**				
FT4, TSH	98	80	67	43
Haemoglobin, platelets	3	3	3	5
Leucocytes neutrophils	4	3	4	4
Liver tests	17	11	17	17
Sodium	1	1	1	3
Potassium	1	1	1	3
Creatinine	1	1	1	3
Calcium	1	1	1	5
HbA1c	6	6	6	6
TRAb	97	97	97	24
TSI (in 1% of patients)	0·2	0·2	0·2	0·2
FT3	4	4	9	9
Calcium (corrected)	0	0	0	14
25-OH-vitamin D3	0	0	0	22
PTH	0	0	0	26
**TOTAL cost**	**235**	**210**	**209**	**185**

FT3, free triiodothyronine; FT4, free thyroxine; HbA1c, glycated haemoglobin; PTH parathyroid hormone; SEK, Swedish krona; TRAb, anti-TSH receptor antibody; TSH thyroid-stimulating hormone; TSI, thyroid-stimulating immunoglobulin; USD, United States dollar.

Table S5: Average cost of laboratory tests before and after introduction of new guideline in USD

Laboratory test	Average cost of tests (Euro)			
	ATD monotherapy	ATD block and replace	RAI	Tx
**Before introduction of new guideline**				
FT4, TSH	78	61	61	39
Haemoglobin, platelets	2	2	2	5
Leucocytes/neutrophils	13	10	5	6
Liver tests	56	46	20	25
Sodium	1	1	1	3
Potassium	1	1	1	3
Creatinine	1	1	1	3
Calcium	1	1	1	5
TRAb	88	88	88	22
FT3	4	4	8	8
Calcium (corrected)	0	0	0	13
PTH	0	0	0	24
**TOTAL cost**	**247**	**217**	**190**	**155**
**After introduction of new guideline**				
FT4, TSH	89	72	61	39
Haemoglobin, platelets	2	2	2	5
Leucocytes neutrophils	3	2	3	3
Liver tests	15	10	15	15
Sodium	1	1	1	3
Potassium	1	1	1	3
Creatinine	1	1	1	3
Calcium	1	1	1	5
HbA1c	6	6	6	6
TRAb	88	88	88	22
TSI (in 1% of patients)	0·2	0·2	0·2	0·2
FT3	4	4	8	8
Calcium (corrected)	0	0	0	13
25-OH-vitamin D3	0	0	0	20
PTH	0	0	0	24
**TOTAL cost**	**214**	**191**	**190**	**169**

FT3, free triiodothyronine; FT4, free thyroxine; HbA1c, glycated haemoglobin; PTH parathyroid hormone; SEK, Swedish krona; TRAb, anti-TSH receptor antibody; TSH thyroid-stimulating hormone; TSI, thyroid-stimulating immunoglobulin.

Table S6: Average cost of laboratory tests before and after introduction of new guideline in Euro

Thyroid nurse	1·9 hours extra
TSI use	1% of cases
Preoperative 25-OH vitamin D3 analysis	In 23·4% of patients (after Sjölin et al. 2019)*
Calcium/vitamin D treatment	In 25% of those operated on
GREAT+ score	In 50% of all patients at diagnosis

*Sjölin G, Holmberg M, Törring O, et al. The long-term outcome of treatment for Graves' hyperthyroidism. *Thyroid*. 2019; **29**: 1545–57. GREAT, Graves' Recurrent Events After Therapy; TSI, thyroid-stimulating immunoglobulin.

Table S7: Estimated new changes after introduction of new guideline
